# Mendelian randomization in SLEEP: avoiding pitfalls with MR-SLEEP guidelines

**DOI:** 10.1093/sleep/zsaf054

**Published:** 2025-03-11

**Authors:** Daniel S Evans, Allan Pack, David Gozal, Katie L Stone

**Affiliations:** Department of Epidemiology and Biostatistics, University of California, San Francisco, CA, USA; California Pacific Medical Center Research Institute, San Francisco, CA, USA; Perelman School of Medicine, University of Pennsylvania, Philadelphia, PA, USA; Joan C. Edwards School of Medicine, Marshall University, Huntington, WV, USA; Department of Epidemiology and Biostatistics, University of California, San Francisco, CA, USA; California Pacific Medical Center Research Institute, San Francisco, CA, USA

## Introduction

**Figure 1.**
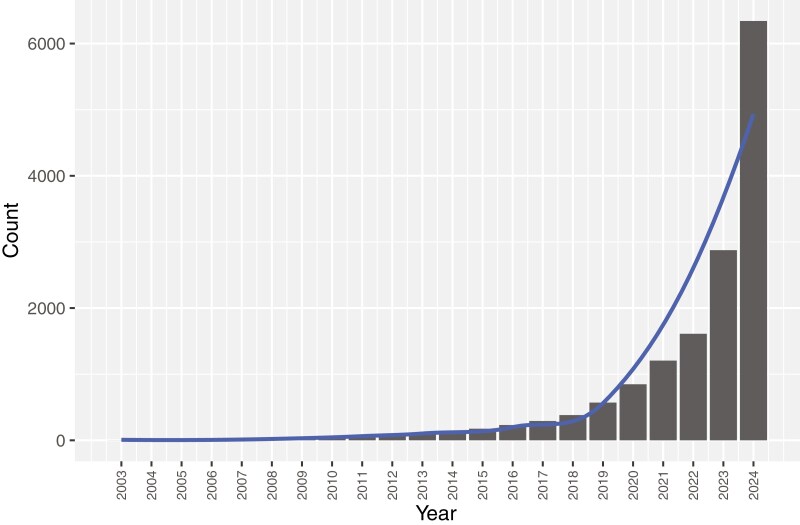
Frequency of PubMed citations for “mendelian randomization” per year.

Since the seminal articles by George Davey Smith and Shah Ebrahim [1] and Martijn Katan [2], there has been an explosion in the number of publications using the statistical analysis approach of Mendelian randomization (MR) (Figure 1). The steep increase in MR publications coincided with genome-wide association study (GWAS) results becoming readily available and hosted by publicly available knowledge portals and catalogs [3–5]. In addition, the development of well-designed and easy-to-use software applications dedicated to MR analyses further spurred interest in these studies [6]. The remarkable rise in the number of MR publications is for good reason, as MR can aid in the identification of causal associations by anchoring traits to genetic variants that are largely immune to the influence of confounding factors. However, MR studies must be done appropriately with careful consideration of the potential pitfalls of this method, particularly since MR makes key assumptions that when violated can threaten conclusions about causality.

Guidelines for the proper implementation of MR, such as STrengthening the Reporting of OBservational studies in Epidemiology using Mendelian Randomization (STROBE-MR), have raised the quality bar for MR studies [7]. However, sleep and circadian research presents unique challenges that require additional considerations. Some of these challenges have been noted previously, particularly focusing on genetic instrument selection [8]. We have observed other common pitfalls in MR manuscripts submitted to SLEEP that typically lead to their rejection. Here, we review the MR method, assumptions required for the proper use of MR, key considerations when performing MR in sleep and circadian research, and how MR in sleep and circadian research can often violate the third MR assumption (the exclusion restriction). We conclude with a supplement to the STROBE-MR guidelines, which we term MR-SLEEP, aimed at addressing the particular challenges of applying MR in our field. The scope of our guidelines includes one and two-sample MR and extensions of MR that rely on the three well-known MR assumptions. To ensure that MR studies submitted to the journal SLEEP are of sufficient quality, manuscript submissions employing the MR method will need to be accompanied by a completed MR-SLEEP checklist and STROBE-MR checklist in order to be considered for peer review.

## Mendelian Randomization Method, Assumptions, and Usage

MR is a form of instrumental variable (IV) analysis using genetic variants. IV analysis, with or without genetic variants, can be used to determine whether an exposure is causally associated with an outcome by leveraging a third variable, an instrument. A valid instrument is associated with the exposure, affects the outcome only through its effect on the exposure, and there are no confounders of the IV and the outcome. To avoid potential confounding, IV analysis takes advantage of an instrument that is randomly assigned to individuals in the study population [9]. For example, an IV analysis used the encouragement to stop smoking as an instrument for smoking behavior to study the effect of smoking in pregnant women on a child’s birth weight [10]. Smoking cessation encouragement was considered to have an effect on maternal smoking behavior and to only affect childbirth weight through a change in maternal smoking.

**Figure 2.**
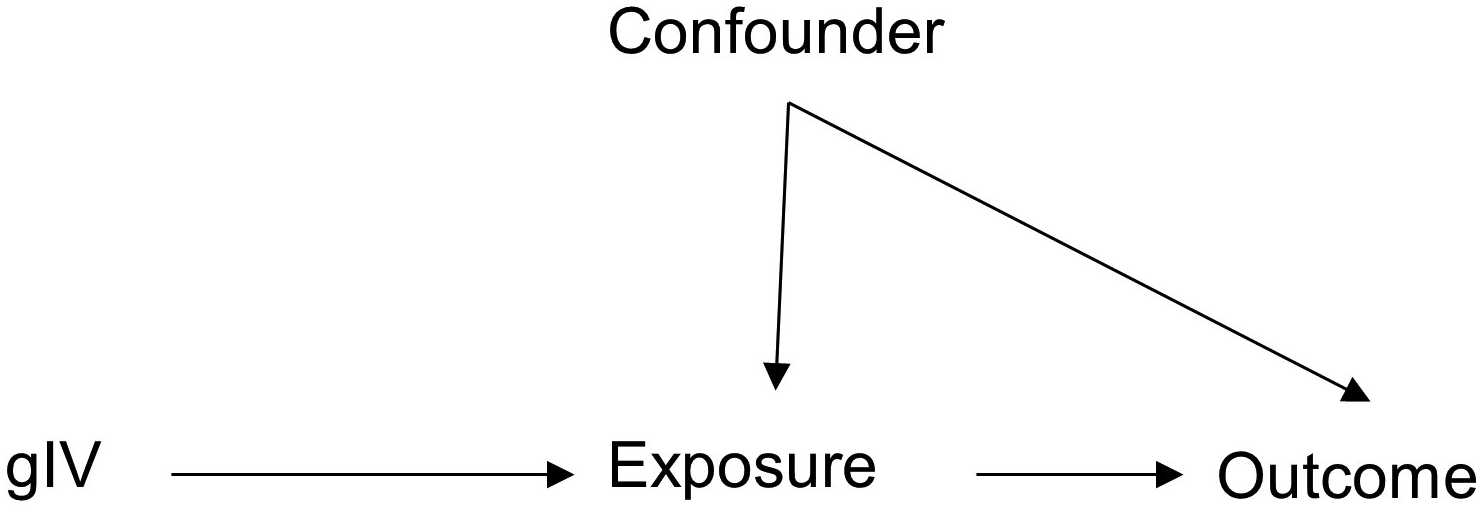
MR framework.

MR is a special case of IV analysis that takes advantage of Mendel’s law of segregation (alleles at a single locus separate during meiosis) and independent assortment (alleles at unlinked loci are inherited independently of one another) by using genetic variants as instruments for traits (Figure 2). The inheritance of trait-associated genetic variants can be conceptualized as a random assignment at birth to a genetically mediated lifetime exposure to an altered level of a particular trait, which can then be tested for an association with an outcome of interest. Genetic variants can serve as excellent instruments because alleles at a locus are randomly inherited from each parent, and if a genetic variant is associated with a trait, that variant can serve as an unconfounded indicator of levels of that trait [11]. As an example, MR using genetic variants associated with plasma low-density lipoprotein cholesterol (LDL-C) levels demonstrated a causal association between lower plasma LDL-C and lower risk of coronary heart disease (CHD) [12]. In the context of sleep and circadian disorders, MR manuscripts submitted to SLEEP have typically examined whether genetic variants associated with sleep and circadian rhythm traits are also associated with health outcomes.

MR makes three statistical assumptions that are closely related to the criteria for valid IVs outlined above [13]. Following the naming and numbering of MR assumptions from Sanderson et al. [14], the first assumption termed the relevance assumption, states that a genetic instrumental variable (gIV) is associated with the exposure, which is depicted by an arrow connecting gIV to exposure in Figure 2. This assumption is typically addressed by only using gIVs associated with an exposure at the genome-wide significance threshold (*p* ≤ 5 × 10^-8^). Additionally, the *F*-statistics of gIV-exposure associations are calculated to identify and remove potential weak instruments. The *p*-value threshold and *F*-statistics do not ensure a causal genetic association with the exposure. In fact, a trait-associated genetic variant is often not the causal variant but is instead in linkage disequilibrium with causal variants. The second MR assumption termed the exchangeability assumption, states that the gIV is independent of all confounders of the exposure-outcome relationship, represented by the absence of an arrow between the gIV and confounder(s) (Figure 2). The third MR assumption, termed the exclusion restriction, states that the gIV does not affect the outcome other than through the exposure, and the gIV does not affect any other trait that has a downstream effect on the outcome. The exclusion restriction assumption is represented by the absence of a single arrow directly connecting the gIV to the outcome (Figure 2) [13, 14].

Many approaches and software packages are available to effectively perform MR, and we do not require the exclusive use of any particular method or software implementation for manuscripts submitted to SLEEP. The vast majority of MR studies submitted to SLEEP take the two-sample MR (2SMR) approach using the well-designed TwoSampleMR R package [6], so we describe this approach to orient the reader. However, MR studies submitted to SLEEP are not required to use this particular method or software. The 2SMR approach is easy and convenient to perform because it makes use of trait associations with genetic variants, typically single nucleotide polymorphisms (SNPs), from publicly available GWAS results without the need for individual-level data. In 2SMR, separate study samples are used to estimate SNP-exposure associations and SNP-outcome associations. In this design, gIVs for an exposure can be selected from robust SNP associations from the largest GWAS of that trait, even if the outcome was not measured in those same samples. This is in contrast to one-sample MR, which typically limits the sample size because of the requirement for the exposure and outcome to be measured in the same individuals [15]. The 2SMR approach also has the advantage of minimizing the effect of the weak instrument bias. Weak instrument bias in one-sample MR is biased towards the confounded observational association, but weak instrument bias in 2SMR is biased to the null, making 2SMR a conservative approach [15, 16].

The first step of 2SMR is to identify a GWAS conducted on the exposure of interest, and then extract genome-wide significant (*p* ≤ 5 × 10^-8^) variant associations. The only information needed is the SNP identifier (typically a dbSNP rs number), effect estimate (beta for continuous outcome or log odds ratio for binary outcome), standard error of the effect estimate, and the effect allele (effect estimate reflects the trait association with higher dosage of the effect allele) [17]. Then, these same genetic variants are extracted from a GWAS conducted on the outcome of interest, regardless of whether the variants are associated with the outcome at the genome-wide significance level. In the case of a continuous exposure and outcome, the betas of the gIV-outcome association are regressed on the betas of the gIV-exposure association, and the slope represents the causal estimate between the exposure and outcome. The slope indicates whether SNPs with larger effect estimates for the exposure also have larger effect estimates for the outcome [11].

The development of statistical tests for MR is an active area of research, with new methods being published frequently. Some of the commonly used MR tests are reviewed here. The fixed-effect inverse variance weighted method is a very simple and straightforward approach, but it is not robust to violations of MR assumptions, so it assumes all of the gIVs are valid [18]. In contrast, the weighted median provides a consistent estimate even if only 50% of the weight comes from valid gIVs [19]. Mendelian randomization using the robust adjusted profile score (MR-RAPS) can accommodate weak instruments, but MR-RAPS makes the additional assumption that gIVs display balanced pleiotropy, which is often not the case [20]. Multiple tests are designed to be robust to violations of the exclusion restriction assumption, such as MR-Egger [21], Mendelian randomization pleiotropy residual sum and outlier (MR-PRESSO) [22], and multivariable MR (MVMR) [23–26]. We describe these methods and the additional assumptions that they make in the section “Violation of the third MR assumption (exclusion restriction).”

Diagnostic and sensitivity analyses, such as the heterogeneity test [27], leave-one-out (LOO) analysis, and funnel plots [6], assess the likelihood of violations of MR assumptions and complement different MR methods. The 2SMR R package simplifies MR analysis by providing functions to perform many of these statistical tests and sensitivity analyses [6].

## Special Considerations for MR in Sleep and Circadian Research

### Validated or objective measurements

Sleep can be viewed as multi-dimensional, encompassing continuous measures (e.g. sleep duration, continuity, regularity, and timing) as well as specific disorders (e.g. insomnia, sleep-disordered breathing, narcolepsy, and restless legs syndrome). Circadian rhythm governs the timing of sleep–wake cycles, as well as other activities throughout the day and night. Circadian rhythm, especially when misaligned with the environment, can also impact the amount and quality of sleep. Given the complexity of sleep, it is not surprising that single items from questionnaires often fail to accurately capture sleep phenotypes. Even a seemingly easy-to-measure trait like sleep duration can differ based on objective and subjective measurements, may change in response to health changes or other stressors, or may result from one or more unmeasured sleep disorders. In a population-based study of middle-aged adults, there was only a moderate correlation between objectively measured sleep duration assessed using wrist-worn actigraphy and subjective sleep duration estimated with the following, “During the past month, how many hours of *actual sleep* did you get at night? (This may be different from the number of hours you spend in bed.) On weekdays? On weekends?” Individuals tended to overestimate their sleep duration, and there was a larger overestimate with shorter objectively measured sleep duration, indicating differential measurement error that is an especially challenging bias for which to statistically adjust [28].

At the journal SLEEP, our priority is to publish research that advances our understanding of sleep and circadian rhythms, as well as their contributions to health and disease. To that end, manuscripts published in SLEEP are typically based on validated and/or objective measurements of sleep and circadian rhythm. For MR studies in our field, this implies that gIVs should be based on genome-wide significant genetic associations from GWAS conducted with validated or objective measurements of sleep or circadian rhythm to ensure that gIVs are predictive of the trait of interest.

We recognize that GWAS conducted with validated sleep measurements generally have smaller sample sizes and result in fewer genome-wide significant genetic associations [8, 29]. However, the journal SLEEP puts forth the position that conducting MR with fewer genetic instruments associated with validated measurements is favorable to conducting MR with more genetic instruments associated with traits that are less certain to be related to sleep and circadian rhythm. Sources of gIVs of validated or objectively measured sleep or circadian rhythm traits/disorders include GWAS of obstructive sleep apnea traits [30, 31], accelerometer-based sleep duration and sleep efficiency [32], restless legs syndrome [33], and narcolepsy [34]. This is a non-exhaustive list, and we welcome MR manuscript submissions using gIVs derived from well-conducted GWAS of validated or objective measures of sleep and circadian rhythm traits. A measurement is typically validated by testing for internal homogeneity, test–retest reliability (consistency), and validity (comparison with a gold standard), as was performed for the Pittsburgh Sleep Quality Index [35]. The Insomnia Severity Index has been validated using similar approaches [36, 37].

Validated measures of sleep are not typically found in large sample sizes in biobank studies, so understandably, many GWAS of sleep traits have been conducted using single items from questionnaires [29]. As a consequence, the vast majority of MR papers focusing on sleep and circadian rhythm traits rely on gIVs that might not accurately reflect the trait intended to be studied. MR studies of sleep duration often rely on gIVs from a GWAS of self-reported sleep duration conducted within the UK Biobank. The self-report was in response to the question, “About how many hours of sleep do you get in every 24 h? (please include naps).” Responses were in hour increments. Sleep duration was treated as a continuous variable and also categorized as short (6 h or less), normal (7 or 8 h), or long (9 h or more) [38]. Objectively measured sleep duration from actigraphy devices was also available from 85 449 UK Biobank participants, a subset of the initial GWAS study population, and to the authors’ credit, genetic associations shared across self-report and objective sleep duration were assessed. Of the 78 genome-wide significant genetic associations with self-reported sleep duration, only five were replicated with objective sleep duration with the same effect direction and multiple testing corrected for 78 tests [38]. With so few of the genetic associations with self-reported sleep duration replicating for the same trait that was objectively measured, even among participants of the same study population, it cannot be assumed that gIVs of self-reported sleep duration accurately predict sleep duration measured objectively.

Another commonly used source of gIVs for MR is a GWAS of chronotype conducted in the UK Biobank using a single item from a questionnaire [39]. Participants were asked “Do you consider yourself to be?” with one of six possible answers: definitely a morning person, more a morning than evening person, more an evening than a morning person, definitely an evening person, do not know, or prefer not to answer. Do not know was coded as zero, midway between definitely morning and definitely evening. Prefer not to answer was coded as missing [39]. This single question is not a validated measure of chronotype, and it cannot be assumed that genetic associations with this trait accurately reflect circadian rhythm or rest-activity rhythm.

### Scientific rationale

With widely available GWAS results and easy-to-use MR software, MR results can be easily produced that are statistically significant but are not grounded in a plausible hypothesis that links sleep and circadian rhythms to the outcome of interest. Without a credible scientific rationale, false positives can easily be generated. MR relies on genetic associations with traits in humans; thus, a scientific rationale for an MR study should include evidence from human clinical research studies. MR is most likely to contribute to the pool of existing knowledge when there is already evidence for an association, but either the effect direction or the causality of the association is uncertain. A well-conducted MR study can help to resolve uncertainty in a research topic by providing a means to substantially reduce the influence of confounding.

We recognize that not all MR studies examine the relationship between a single exposure and a single outcome. Genomic discovery MR studies can seek to identify potentially causal associations between a large collection of biomolecules and a particular sleep or circadian rhythm trait. Genomic discovery MR studies should provide a rationale supporting the role of the biomolecule class under study (protein, metabolite, etc.) and the sleep trait being examined as the outcome. MR can also be used to follow up non-human molecular findings, but authors still need to provide the rationale for performing MR in humans with the candidate molecular exposure and sleep trait.

### Violation of the third MR assumption (exclusion restriction)

**Figure 3.**
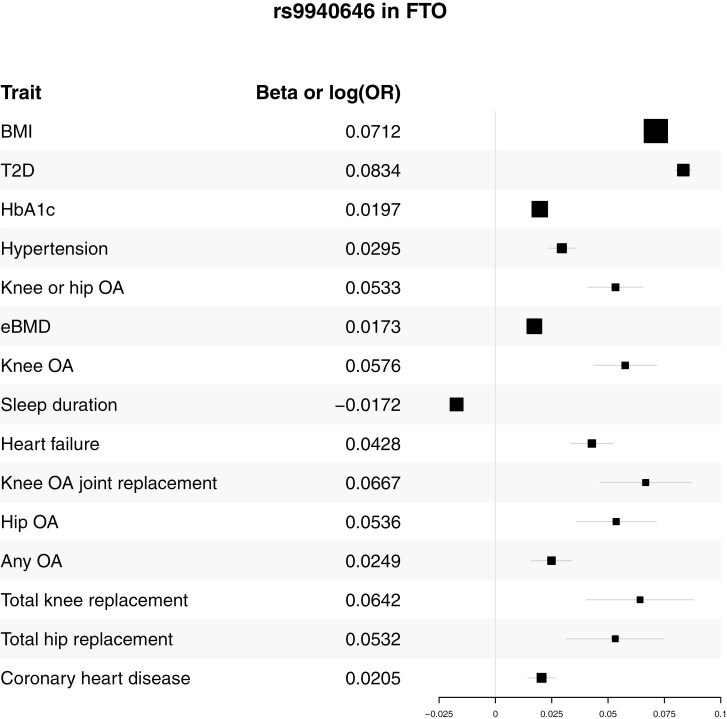
Trait associations with rs9940646 from the T2DKP. Effect size along the *x*-axis, point estimate size proportional to 1/*SE*, 95% confidence intervals shown.

The third MR assumption, the exclusion restriction, states that a gIV does not affect the outcome other than through the exposure, and a gIV does not affect any other trait that has a downstream effect on the outcome [14]. Pleiotropy (genetic variants with multiple independent trait associations) can result in a violation of the exclusion restriction assumption. Even gIVs based on GWAS of validated, objectively measured sleep or circadian rhythm traits should be checked for violations of the third MR assumption. Many traits and common chronic conditions are associated with sleep disruption [40], and genetic associations with these chronic conditions can indirectly result in genetic associations with sleep and circadian rhythm traits. Case in point, rs9940646 in the *FTO* gene is one of the five SNPs associated with self-reported sleep duration that replicated with objectively measured sleep duration [38]. Genetic variants in the *FTO* gene region were the first genetic associations discovered in GWA studies of obesity, and the associations are robust among diverse populations [41]. From a biological understanding of this genetic association, rs9940646 is primarily associated with obesity, and only secondarily associated with sleep duration. A sleep duration gIV residing in the *FTO* gene region raises the obvious concern that this SNP can induce apparent MR associations between sleep duration and health outcomes that merely reflect rs9940646’s association with obesity. As expected, a lookup of rs9940646 in the Type 2 Diabetes Knowledge Portal (T2DKP) [5] revealed significant genetic associations with sleep duration, obesity-related traits, and outcomes that could plausibly be related to obesity, such as hypertension, osteoarthritis, bone mineral density, heart failure, and CHD (Figure 3). For MR studies of sleep duration and outcomes in which obesity is a risk factor, rs9940646 would clearly violate MR’s exclusion restriction assumption. This concern is not limited to gIVs for sleep duration. In a GWAS of sleep apnea, SNPs in *FTO* were far and away the most significantly associated, and the genetic associations were no longer significant upon adjustment for body mass index (BMI) [42].

Multiple approaches exist to identify possible violations of the exclusion restriction MR assumption, and then to subsequently obtain MR associations that can be robust to this class of assumption violations. Funnel plot asymmetry and tests of heterogeneity can be used to detect potential pleiotropic gIVs that violate the exclusion restriction assumption [6]. Evidence of a non-zero Y-intercept from the MR-Egger test can also indicate a violation of the exclusion restriction assumption [21]. These statistical and graphical tests do not incorporate subject-matter knowledge about the SNPs used as gIVs and whether they are associated with risk factors for the outcome of an MR study. To incorporate subject-matter knowledge, one can examine whether each SNP from a multi-SNP MR instrument is associated with risk factors of an outcome in a particular MR study [6]. This is typically performed by searching published GWAS results using phenoscanner or the large collection of disease-focused knowledge portals within the knowledge portal network [5, 43]. The knowledge portal network (kp4cd.org) is particularly easy to use for a wide range of scientific users. Our evaluation of the SNP in *FTO* associated with subjective and objective sleep duration in Figure 3 was performed using the knowledge portal network.

If there appears to be evidence of a violation of the exclusion restriction MR assumption, there are multiple MR approaches designed to be robust to this violation, but these methods also come with additional statistical assumptions. MR-Egger allows for a non-zero Y-intercept and is designed to be robust to violations of the exclusion restriction assumption [21]. However, MR-Egger makes an additional assumption, the Instrument Strength Independent of Direct Effect (InSIDE) assumption [44]. Moreover, a single outlying variant can influence MR-Egger’s slope and intercept estimates [44]. MR-PRESSO is also designed to be robust to violations of the exclusion restriction assumption. However, like other outlier-detection methods, MR-PRESSO requires at least 50% of the gIVs to be valid and without horizontal pleiotropy, and in addition assumes balanced pleiotropy and a valid InSIDE assumption [22]. Another approach designed to be robust to pleiotropy, that is, violation of the exclusion restriction assumption, is MVMR. There are multiple implementations of MVMR [23–26], but they all make additional assumptions. Most notably, MVMR assumes that exposures must be strongly predicted by gIVs conditional on the other exposures included in the estimation [26]. For example, in a multivariable MR model with sleep duration and BMI as exposures, the set of SNPs used as gIVs must be able to strongly predict sleep duration once the association between the SNPs and BMI has been accounted for. Information on conditional SNP association is not always available from a standard GWAS, but in the case of sleep apnea, BMI-adjusted SNP associations were reported [42]. Users of MVMR and other methods designed to be robust to pleiotropy must provide evidence supporting the validity of these additional assumptions. While these methods can be very useful, subject-matter knowledge is not leveraged.

Subject-matter knowledge can be incorporated into sensitivity analysis by combining GWAS database searches with LOO analysis. LOO is an approach designed to evaluate if the MR estimate is driven or biased by a single SNP by sequentially dropping one SNP at a time and re-estimating the overall MR association [6]. If a dropped SNP results in a large change in the MR estimate and the SNP is also associated with a risk factor for the outcome other than the instrumented exposure, the MR association is likely invalid due to a violation of the third MR assumption. LOO analysis can miss violations of MR’s third assumption if more than one SNP from a multi-SNP instrument is associated with risk factors for the outcome. Thus, performing sensitivity analysis removing all such SNPs would be the most conservative approach (leave-many-out).

## MR-SLEEP Guidelines

To address the three key challenges facing MR in sleep and circadian rhythm research, we introduce MR-SLEEP guidelines. Our guidelines are composed of the three items listed below, and can be found in a fillable Word document (MR-SLEEP-checklist-fillable.docx) on our submission site and in the Supplementary Material of this editorial. The scope of our guidelines includes one and two-sample MR and extensions of MR that rely on the three well-known MR assumptions. Genomic discovery MR studies considering a sleep trait as an outcome and potentially thousands of biomolecules as exposures only need to perform sensitivity analyses (LOO and leave-many-out) for MR associations passing multiple test correction. In addition to the requirement to perform LOO and leave-many-out for multi-SNP instruments, authors may also choose to add pleiotropy-robust methods in addition to, not instead of, LOO and leave-many-out. If authors choose to perform additional pleiotropy-robust methods, authors then must assess the validity of the additional assumptions made by these methods and discuss reasons for potential differences with LOO and leave-many-out.

1. Describe how the sleep and/or circadian rhythm trait was measured or diagnosed in the GWAS that identified the gIVs.
2. Describe the scientific rationale for this MR study based on evidence from human clinical research studies. Genomic discovery MR studies of biomolecules and a particular sleep trait should provide a rationale supporting the role of the biomolecule class under study (protein, metabolite, etc) and the sleep trait being examined as the outcome. MR studies following up on non-human results should provide the rationale for performing MR in humans with the candidate molecular exposure and sleep trait.
3. Assess violation of the exclusion restriction MR assumption with the following four steps. (1) Provide a list of widely accepted or likely potential predictors of the outcome under investigation. (2) Provide a spreadsheet (not pdf) of the gIVs used and their association with predictors of the outcome from a lookup from a GWAS database, such as the knowledge portal network (kp4cd.org). (3) If more than one variant is used as a gIV, provide LOO analysis plots and discuss whether any dropped SNPs that affect the overall MR estimate are also associated with predictors of the outcome other than the primary exposure. (4) Repeat the MR analysis after removing all gIVs that are genome-wide significantly (*p* ≤ 5 × 10^-8^) associated with potential predictors of the outcome other than the primary exposure (leave-many-out).

MR papers submitted to the SLEEP journal must include a completed MR-SLEEP checklist in addition to the STROBE-MR checklist. With these new guidelines, we hope that MR will be used to provide meaningful contributions to the field, and we invite submissions of MR manuscripts to the journal SLEEP.

## Supplementary Material

Supplementary material is available at *SLEEP* online.

zsaf054_suppl_Supplementary_Materials

## Data Availability

No new data were generated or analysed in support of this research.
