# Supplementary material for: Mendelian randomization in SLEEP: avoiding pitfalls with MR-SLEEP guidelines
Source: Sleep. 2025 Mar 11;48(6):zsaf054. doi: 10.1093/sleep/zsaf054 (PMC12163116; doi:10.1093/sleep/zsaf054)
Supplement: zsaf054_suppl_Supplementary_Materials [file zsaf054_suppl_supplementary_materials.docx]

**MR-SLEEP checklist of required items to address in Mendelian Randomization manuscripts submitted to SLEEP**

| **Item No.** | **Checklist item** | **Page No.** | **Relevant text from manuscript** |
| --- | --- | --- | --- |
| 1 | Describe how the sleep and/or circadian rhythm trait was measured or diagnosed in the GWAS that identified the gIVs. |  |  |
| 2 | Describe the scientific rationale for this MR study based on evidence from human clinical research studies. Genomic discovery MR studies of biomolecules and a particular sleep trait should provide a rationale supporting the role of the biomolecule class under study (protein, metabolite, etc) and the sleep trait being examined as the outcome. MR studies following up non-human results should provide the rationale for performing MR in humans with the candidate molecular exposure and sleep trait. |  |  |
| 3 | Assess violation of the exclusion restriction MR assumption with the following four steps. 1) Provide a list of widely-accepted or likely potential predictors of the outcome under investigation. 2) Provide a spreadsheet (not pdf) of the gIVs used and their association with predictors of the outcome from a lookup from a GWAS database, such as the knowledge portal network (kp4cd.org). 3) If more than one variant is used as a gIV, provide LOO analysis plots and discuss whether any dropped SNPs that affect the overall MR estimate are also associated with predictors of the outcome other than the primary exposure. 4) Repeat the MR analysis after removing all gIVs that are genome-wide significantly (P ≤ 5x10^-8^) associated with potential predictors of the outcome other than the primary exposure (leave-many-out). |  |  |
